# Annotations

**Published:** 1894-12-15

**Authors:** 


### ANNOTATIONS.

### Religion in Hospitals.

Although the origin of hospitals is due to religious communities, the spirit of tolerance, and a broader minded Christianity, has long since banished the rule that a hospital patient should conform to any particular creed; proselytising, indeed, has come to be regarded as an odious stigma on any institution. The most carefully organised hospitals guard against the possibility of such an accusation, by framing rules to provide against it, and seeing that they are carried out. But even in spite of all due care, unkindly whispers will arise. Such has been the case at the Edinburgh Royal Infirmary. Unsubstantiated and anonymous accusations of proselytising within its walls have been made. Yet all that a wise management can do has been done to prevent any interference with the religious belief of the patients. The rules are most stringent, forbidding the chaplain and his assistants even to converse with a patient on matters of religion if any objection is made. All visitors are bound by the same rules; and no tracts may be distributed without the chaplain's sanction. Any clergyman may visit one of his flock, and thus security is assured. The public show a regrettable tendency to rush into print, when the smallest point in the armour of a hospital is assailed, without inquiry from the authorities. If the assailants of the Edinburgh Royal Infirmary had studied the rules of that institution, they would have seen that any patient could have silenced interference in religious matters by appealing to the authorities whose rules the visitor was breaking. The accusation applied to a visitor, as neither the chaplain nor his assistant had any knowledge of the matter when referred to at the last meeting of the hospital.

### The Disappearance of the Tadpole's Tail.

Ever since there were tadpoles men have known that as the legs become developed the tail disappears. Not until within the last few years have they cared to look for the reason or to investigate the *modus operandi* of this curious and interesting phenomenon. To the biologist the disappearance of a structure by apparently normal and harmless processes is scientifically important, as every fact and process in nature is important. To the pathologist such phenomena are of added significance, since they cannot but help him to understand the degenerative and reparative processes with which he is familiar in the human organism, and which constitute no small part of the basis of the healer's assurance of success in his work. Dr. Joseph Griffiths, following in the footsteps of Metchnikoff, Barfurth, and others, has undertaken a research into the physiological processes which accompany or bring about the gradual and complete disappearance of the tail of the tadpole. Essentially the process seems to consist in the fatty degeneration of the muscular and other structures of the tail, and the absorption of the fattily degenerated products by phagocytes, newly developed for the purpose. The phagocytes, in their turn, after having accomplished their destiny, degenerate structurally become fluid, and passing along the course of the lymph current, enter the blood and furnish nutriment to the whole bodily organism. Dr. Griffiths is not quite able to confirm Metchnikoff's view that the case is one of simple fatty degeneration followed by phagocytosis. He finds no structures in the disappearing tail resembling leucocytes or ordinary phagocytes. What he does find is a series of cells, which he calls sarcomites, developed within the sarcolemma from the proliferating nuclei, and which, in the first stage, are small, with large, round nuclei and finely granular protoplasm, resembling, but not being, leucocytes; in the second stage these are of intermediate size, capable of amœboid movements, and probably of phagocytic action; and, in the third, are large, globular or irregular in shape, and appear laden, or "over-laden," with fat granules and globules. It would seem, then, that we have not here a phagocytic action identical with that of ordinary phagocytosis—that is, a series of phagocytic cells meeting and destroying some foreign deleterious substance casually introduced into the body—but a development of cells from local structures (the muscle nuclei) whose one business in life it is to devour the substance in which they origi nate The subject is exceedingly interesting, and we are glad to note that Dr. Griffiths promises the profession further communications.

### Experiments in Immunisation.

Will it ever be possible to build up a barrier of absolute immunity against every variety of bacillary disease? Theoretically it should. In practice we find that the more profoundly the question of immunisation is studied the greater are the difficulties which stand in the way of rendering our theories concrete. A series of very interesting experiments have recently been made by Dr. W. S. Melsome and Mr. Louis Cobbett with the object of rendering animals immune against erysipelas. *Streptococcus erysipelatis* is a bacillus readily capable of cultivation on beef broth. But after a few days it is found that it ceases to grow on the broth, and dies. Why is this? Drs. Melsome and Cobbett find that the reason why the streptococcus ceases to grow on beef broth is that the pabulum suitable for its growth is exhausted. They deny altogether that the bacillus has secreted a toxin which has caused its own death. This theory of the exhaustion of pabulum would seem to be confirmed by the fact that colonies of the streptococcus often contain a few living bacilli after most of them are dead, and if those which remain alive are sown on solid media, they give rise to new colonies at once. Animals inoculated with cultures thus produced are rendered locally and generally immune against attacks of erysipelas; but the immunity, both local and general, is of limited duration, and varies with the parts inoculated. The results obtained, though disappointing from the point of view of clinical immunisation, are interesting from the stand-point of science. It is a great point to establish that exhaustion of pabulum is a factor in immunisation. It is also of importance to have demonstrated that immunisation can be rendered more or less complete, locally and generally, by choice of locality for inoculation. A significant fact is that, in the case of erysipelas, the duration of immunity is to be counted only by weeks and days, and though it may run into months, by no means extends to years.

